# Glucose Abnormalities Associated to Prolactin Secreting Pituitary Adenomas

**DOI:** 10.3389/fendo.2019.00327

**Published:** 2019-05-22

**Authors:** Renata S. Auriemma, Dario De Alcubierre, Rosa Pirchio, Rosario Pivonello, Annamaria Colao

**Affiliations:** Dipartimento di Medicina Clinica e Chirurgia, University “Federico II”, Naples, Italy

**Keywords:** hyperprolactinemia, pituitary tumor, dopamine agonists, bromocriptine, cabergoline, glucose metabolism, insulin metabolism, metabolic syndrome

## Abstract

The pathogenesis of obesity and alterations in glucose profile have been linked to PRL excess, as it is reportedly associated with metabolic syndrome in thereabout one third of patients. *In vitro* exposure of pancreatic islet to PRL is known to stimulate insulin secretion and β-cell proliferation, and in turn overexpression of PRL in β-cells increases insulin release and β-cell replication. PRL excess has been found to worsen glucose profile because it reduces glucose tolerance and induces insulin resistance either in obese and non-obese patients. To note, pancreatic β-cells and adipocytes widely express dopamine receptors type 2, and dopamine has been hypothesized to play a key role as modulator of insulin and adipose functions. The dopamine agonists bromocriptine and cabergoline significantly improve abnormalities in glucose profile and reduce the prevalence of metabolic syndrome in a remarkable proportion of patients, regardless of whether body weight and PRL status may change. However, in men with hyperprolactinemia complicated by hypogonadism, testosterone replacement can ameliorate insulin resistance and abnormalities in glucose metabolism. Therefore, in patients with PRL-secreting pituitary adenomas control of PRL excess by dopamine agonists is mandatory to improve glucose and insulin abnormalities.

## Introduction

PRL excess may promote the development of disorders in glucose and insulin metabolism (1–7). Indeed, hyperprolactinemia has been related to the pathogenesis of impaired glucose tolerance and hyperinsulinemia up to overt insulin resistance (8–14), as demonstrated by the evidence that high PRL levels promote the increase of the surrogate index of insulin resistance (HOMA-IR, 11–13) and the reduction of the surrogate index of insulin sensitivity (14), either in obese and lean patients. PRL excess induces the functional blockade of dopaminergic tone, which in turn may be accounted among factors implied in the pathogenesis of hyperphagia and weight gain seen in patients with hyperprolactinemia, so contributing to obesity (15–21). On the other hand, *in vitro* studies in rats and cell lines have clearly reported that insulin secretion and β-cell proliferation are enhanced by the exposure of isolated pancreatic islet to PRL (3, 6), and overexpression of PRL in β-cells has been demonstrated to inappropriately rise insulin secretion and release, also promoting β-cell replication (7). However, animal and human pancreatic β-cells (22) and human adipocytes (23) also express dopamine receptors type 2 (D_2_R), suggesting that, aside from PRL, also dopamine may modulate insulin and adipose functions at peripheral level.

The dopamine agonists bromocriptine and cabergoline nowadays represent the treatment of choice for patients with hyperprolactinemia (24, 25). Interestingly, both drugs have been demonstrated to significantly improve glucose profile in diabetic patients independently on PRL status (26–29, 29–35). Particularly, bromocriptine-Quick Release, a quick-release formulation of bromocriptine mesylate, has been found to reduce glucose levels in non-diabetic obese hyperinsulinemic women on a weight maintaining diet (29). Similarly, this drug has been reported to reduce body weight, glycated hemoglobin (HbA_1c_) and fasting plasma glucose in both diabetic and non-diabetic subjects (28, 32). Therefore, bromocriptine-Quick Release has been officially approved by FDA for patients with type 2 diabetes mellitus (33). It is recommended for patients with type 2 diabetes mellitus with mild hyperglycemia either as adjunctive treatment to metformin and sulfonylurea, or as monotherapy in patients with intolerance to such compounds (33). A similar efficacy on HbA_1c_ has been recently demonstrated also for cabergoline in diabetic patients not adequately controlled by other anti-diabetic drugs (35).

In patients with prolactinomas, bromocriptine has been found to significantly improve glucose homeostasis and insulin resistance (17, 18, 36, 37), and to reduce body weight (17, 37). Likewise, in men and non-obese women with prolactinomas treated with cabergoline the percentage of body fat has been found significantly lower than in treatment-naïve patients (38, 39), suggesting that cabergoline may help in minimizing the risk of obesity (38, 39). The improvement in insulin resistance and glucose profile has been described also in patients with prolactinomas receiving therapy with cabergoline (40–43). Such metabolic gain appears to be independent on the degree of reduction in PRL levels, and may be directly attributable to cabergoline dose instead (42, 43). In men with prolactinomas and persistent hypogonadism, accounting for up to 50% of cases (44–46), proper testosterone treatment may enhance the effects of cabergoline by further reducing insulin resistance and metabolic syndrome (47).

This review focuses on the effects of PRL excess and its control by medical treatment with dopamine agonists on abnormalities in glucose profile.

## Effects on Glucose Abnormalities

Several studies (2–7, 22, 47–70) have investigated the link between PRL levels and gluco-insulinemic metabolism. In rodents, PRL is responsible for peculiar changes in pancreatic β-cell mass and function during pregnancy (2–7). Indeed, the pregnancy-related physiological elevation in PRL levels results in increased β-cell mass and enhanced glucose-induced insulin secretion (2, 48, 49), and the expression of PRL receptors is known to grow on both insulin-secreting cell lines and β-cells (5). When isolated pancreatic islet are exposed to PRL *in vitro*, insulin release and β-cell proliferation rise (3, 6), and overexpression of PRL in β-cells promotes the increase in insulin concentrations and the replication of β-cells (7). In rats and humans, PRL enhances β-cell proliferation, insulin gene transcription and glucose-induced insulin secretion (50–60). In neonatal rat islets PRL treatment enhances islet insulin content and early insulin secretion (54–56), also increasing islet sensitivity to glucose (53). This effect may be partly explained by the increased β-cell and liver glucose transporter GLUT2 in membrane as seen in cultured neonatal rat islets (57, 58). In transgenic female mice with hyperprolactinemia, hyperandrogenism, and hyperprogesteronemia induced by overexpression of the human chorionic gonadotropin β-subunit, a 1-week treatment with cabergoline has been found to significantly reduce body weight and to improve dyslipidemia and insulin resistance up to complete normalization of triglycerides and insulin, besides PRL levels (61). Intraperitoneal glucose and insulin tolerance tests demonstrated cabergoline to significantly improve glucose levels and to prevent insulin resistance (61). These findings supported a key role for PRL as a modulator of insulin secretion and action, and suggested the potential use of medical therapy with dopamine agonists to treat metabolic alterations associated to hyperprolactinemia.

In patients with hyperprolactinemia, an increased response of insulin to glucose has been reported during the oral glucose tolerance test (62–64). This condition of hyperinsulinemia has been found not to parallel glucose uptake and utilization in skeletal muscle (64). Moreover, considering that the reduction of serum free fatty acid levels during the oral glucose load has been found smaller in hyperprolactinemic patients as compared to healthy controls, an impaired antilipolytic effect of insulin in patients with PRL excess has been hypothesized (64). However, such metabolic abnormalities may be independent on PRL levels. In a population of 27 men PRL levels have been found to inversely correlate with insulin and HOMA-IR levels and to directly correlate with adiponectin, regardless from patient BMI (65). In biopsies from visceral and subcutaneous adipose tissue of either lean, overweight and obese patients PRL levels resulted to positively correlate with the adipose tissue fitness markers *PPARG, ADIPOQ*, and *GLUT4* (65), suggesting that PRL might act as a regulator of insulin sensitivity and metabolic homeostasis in adipose tissue in humans (65).

Pancreatic β-cells express also dopamine receptors, and their activation by treatment with dopamine agonists has been shown to suppress glucose-stimulated insulin secretion either in rodents (22) and in patients with Parkinson's disease (66–68). Moreover, in patients with Cushing's disease unsuccessfully treated by surgery chronic treatment with cabergoline has been reported to improve gluco-insulinemic profile and insulin resistance, also reducing the prevalence of diabetes mellitus and impaired fasting glucose, and requiring the withdrawal of treatment with anti-diabetic drugs in some cases (69). In diabetic normoprolactinemic patients, bromocriptine and cabergoline have been demonstrated to significantly improve glucose profile (28–35). Bromocriptine-Quick Release has been found to reduce glucose levels and to improve glucose tolerance in both diabetic and non-diabetic subjects (28, 29, 32). Particularly, in a 16-week double blind study in obese patients with type 2 diabetes mellitus (32) bromocriptine has been demonstrated to significantly reduce HbA_1c_, fasting plasma glucose and mean plasma glucose concentration during oral glucose tolerance test, independently on changes in body weight or body composition (32). During the insulin clamp, only patients treated bromocriptine, but not those receiving placebo, have been found to show a significant improvement in total and nonoxidative glucose disposal (32). Similarly, in diabetic normoprolactinemic patients with mild hyperglycemia, defined as a HbA_1c_ level between 7 and 10%, the addition of cabergoline 0.5 mg/week to treatment with ≥2 oral glucose-lowering drugs has been demonstrated to significantly reduce HbA_1c_ levels after 3 months as compared to placebo (35). Adequate metabolic control, defined as a HbA_1c_ level < 7%, was achieved in 65% of patients and in 45% of subjects receiving placebo (35).

In patients with prolactinomas glucose profile and insulin resistance have been reported to improve following treatment with bromocriptine and cabergoline (39, 40, 42, 43, 47) (Table 1). Particularly, in patients treated with bromocriptine percent change in insulin sensitivity has been found correlated to percent decrease in PRL levels and to bromocriptine dose during the euglycemic hyperinsulinemic clamp (37). In patients treated with long-term cabergoline and receiving doses >0.5 mg/week a significant reduction in fasting insulin and HOMA-IR has been reported (42, 43), with no impact of changes in body weight and BMI (43). At the same time, insulin secretion and sensitivity, as well as fasting glucose levels, have been found also to significantly ameliorate after treatment with cabergoline (43), and in turn cabergoline dose has been demonstrated to predict the decrease in fasting insulin (43), supporting the hypothesis that cabergoline may directly modulate insulin secretion and sensitivity (43). Moreover, in men with prolactinomas and consequent hypogonadism, insulin profile significantly improved after long-term treatment with cabergoline, and an additional amelioration was demonstrated after complete testosterone normalization following androgen replacement (47). Interestingly, also in such patients cabergoline dose has been shown to significantly correlate with insulin levels (47), confirming the hypothesis that dopamine may play a key role as direct modulator of insulin secretion (43). Conversely, testosterone levels have been seen significantly correlated with peripheral insulin sensitivity (47).

**Table 1:** Effects of dopamine agonists bromoscriptine and cabergoline on metabolic abnormalities in patients with hyperprolactinemia.

	**Bromocriptine**	**Cabergoline**
Body weight	↓	↓
BMI	↓	↓
Fasting glucose	↓	↓
Fasting insulin	↓	↓
Insulin sensitivity	↑	↑
Insulin resistance	↓	↓
Prevalence of metabolic syndrome	↓	↓

*Arrows indicate the increase or the decrees of metabolic parameters*.

As a consequence of the effects of cabergoline on insulin secretion and peripheral sensitivity, prevalence of metabolic syndrome reduces from 32 to 10% (40, 42, 43, 47) of patients with hypeprolactinemia receiving long-term therapy.

Altogether, these findings have demonstrated the beneficial impact of the treatment with dopamine agonists on gluco-metabolic alterations in patients with prolactinomas, raising the question of whether these compounds might be proposed as valid alternative or adjunctive treatment in diabetic and non-diabetic patients who cannot achieve adequate metabolic control with standard therapies. Based on this evidence, the use of dopamine agonists might be recommended even independently on PRL status, mainly in hypogonadal premenopausal women with microadenomas with no pregnancy desire who may be theoretically treated only with oral contraceptives (71). In such patients, the treatment with dopamine agonists might induce a direct remarkable improvement of gluco-insulinemic profile. The amelioration of glucose homeostasis abnormalities and metabolic syndrome could be even more relevant in women in the postmenopausal period, when weight loss and improved insulin sensitivity might contribute to reduce cardiovascular risk (72). Therefore, the treatment with cabergoline might be maintained in menopausal women regardless from PRL levels in order to retain the beneficial action of dopamine agonists on metabolic profile.

## Author Contributions

RA, DD, and RPir made substantial contributions to review of literature, acquisition of data, and interpretation of results. RA participated in drafting the article. RPiv and AC participated in revising critically the manuscript for important intellectual content. All authors provided critical feedback and helped shape the manuscript. AC gave final approval of the version to be submitted and any revised version.

### Conflict of Interest Statement

AC has been principal investigator of research studies from Novartis, Ipsen, Pfizer, and Lilly, has received research grants from Ferring, Lilly, Ipsen, Merck-Serono, Novartis, Novo-Nordisk and Pfizer, has been occasional consultant for Novartis, Ipsen and Pfizer, and has received fees and honoraria from Ipsen, Novartis, and Pfizer. RPiv has been principal investigator of research studies from Novartis and HRA Pharma, has received research grants from Novartis, Ipsen, Pfizer, Viropharma and IBSA, has been occasional consultant for Novartis, Ipsen, Pfizer, Viropharma, Ferring and Italfarmaco, and received lecture fees and honoraria from Novartis, Pfizer, and Shire. The remaining authors declare that the research was conducted in the absence of any commercial or financial relationships that could be construed as a potential conflict of interest
